# An Atypical Presentation of Terrible Triad Injury in a Healthy 51-Year-Old Female Patient

**DOI:** 10.7759/cureus.79834

**Published:** 2025-02-28

**Authors:** Andrew Lew, Suleman Janjua

**Affiliations:** 1 Sports Medicine, William Carey University College of Osteopathic Medicine, Hattiesburg, USA; 2 Sports Medicine, Ascension Providence Hospital, Southfield, USA

**Keywords:** common extensor tendon, complex elbow dislocation, coronoid process fracture, elbow instability, lateral collateral ligament, radial collateral ligament, radial head fracture, terrible triad injury of the elbow

## Abstract

A left elbow fracture-dislocation in a 51-year-old female patient following a backward fall at home presented with a comminuted radial head fracture, coronoid fracture, and ruptured collateral ligaments. The patient underwent open reduction and internal fixation (ORIF) with a long-arm splint. Post surgery, aggressive rehabilitation was initiated to restore the range of motion (ROM). Compared to previous reports, this case demonstrates successful management of a severe elbow injury with excellent short-term outcomes. This case highlights the occurrence of the terrible triad in a patient without significant comorbidities and a low-energy fall, as well as the importance of early intervention, proper imaging, and interprofessional collaboration in managing such rare presentations.

## Introduction

The terrible triad injury of the elbow is a severe injury pattern involving three key components: elbow dislocation, radial head fractures, and coronoid process fracture [1]. The elbow is stabilized by multiple structures, including lateral and medial ligaments, and the supporting compartments can be categorized by anterior, posterior, lateral, and medial columns. The anterior column includes the coronoid process, brachialis, and the anterior capsule. The posterior column comprises the olecranon process, triceps, and the posterior capsule. The lateral column involves the lateral collateral ligaments (LCL), radial head, and the capitellum. The medial column involves the medial collateral ligament (MCL), coronoid process, and the medial epicondyle. Elbow dislocations can be differentiated as either simple (without fractures) or complex (with fractures and ligament injuries) [1], and by the mechanism of injury from high or low-energy impact onto an outstretched hand, causing valgus and axial compression of the supinated forearm [2]. Roughly 60% of complex dislocations are due to falling from a standing position, a type of low-energy impact injury [3].

Major elbow joint stabilizers can be differentiated as primary and secondary. Primary elbow joint stabilizers include the ulnohumeral joint, the anterior bundle of the MCL, and the LCL complex. The coronoid process is important as it supports the elbow joint and resists varus and posterior movement. Secondary elbow joint stabilizers include the radial head, flexor-pronator, extensor tendon origins, and joint capsule. The radial head acts as a secondary stabilizer to the valgus motion of the elbow. The secondary stabilizers become critical in the presence of disruption to one or more primary structures. However, there is no significant instability in the disruption of secondary stabilizers in the absence of primary stabilizer dysfunction [4].

A link between elbow dislocation, radial head fracture, and LCL complex injury was first introduced in 1962 by Osborne [5]. The combination of a posterolateral dislocation of the elbow joint, radial head fracture, and coronoid process fracture was later coined the "terrible triad" by Hotckiss in 1966 [4,6]. Generally, the terrible triad can be diagnosed with the presence of two of the three but can sometimes include variant-type injuries discussed later. Historically, it has a poor prognosis and high treatment failures with complications such as redislocation, chronic instability, and elbow osteoarthritis [4]. 

Radial head fractures are involved in 30% of elbow injury cases and can be categorized by Mason classification [4]. Mason type I is a nondisplaced fracture, and the approach is generally nonsurgical. Mason type II is a partially displaced fracture requiring open reduction internal fixation. Mason type III is a complete displacement fracture, requiring excision or radial head arthroplasty.

Coronoid process fractures were historically categorized by Regan and Morrey, but most recently by the Mayo classification. Class I Mayo involves only the tip of the coronoid process which is the most common presentation in terrible triad injury, prompting fixation if there is significant elbow instability. Class II Mayo goes down to the anteromedial facet and is uniquely different from terrible triad injuries, and Class II Mayo involves the base. The stability of the elbow is greatly affected by the size of the coronoid fracture and is generally minimal if there is less than ten percent involvement [4,7].

Currently, there are no classifications for terrible triad injuries of the elbow; however, injuries typically result from falls onto an extended arm that cause axial load and valgus stress, disrupting the LCL complex. During pronated falls, the anterior bundle of the MCL usually fails. Supinated falls usually result in simple dislocations. Further, the falls generally described involve motions involving posteromedial and posterolateral with external rotation, or posteromedial with internal rotation. In terrible triad injuries, the most common mechanism is a posterolateral external rotation with the sparing of the MCL. Coronoid fractures commonly occur in posteromedial falls with sparing of the radial head [7].

Workup for a suspected terrible triad of the elbow includes neurovascular assessment and anterior and lateral elbow imaging with the radial head aligned with the capitellum. If dislocation is present, reduction by axial traction to a partially flexed and supinated elbow is critical to reduce pain and injury. Post-reduction computed tomography (CT) aids in helping evaluate for any fragmentation of the bony structures and guide treatment [7].

Due to the complexity of the injury, outcomes are traditionally poor and usually associated with stiffness, pain, arthritis, and joint instability [2]. Untreated elbow instability leads to significant post-traumatic osteoarthritis [5]. Therefore, recognizing the signs such as pain, swelling, elbow instability, and deformity early and confirming the diagnosis by imaging is crucial for management [8]. Surgical intervention helps restore the stability of the humeroulnar and humeroradial joints and reduces the chances of long-term joint stiffness or disability [8,9]. Surgical goals aim to stabilize the radiocapitellar joint by repairing the lateral structures, including the radial head and lateral collateral ulnar ligament [5]. After the stabilization of the elbow, the primary goal is to allow for early mobilization [10].

This case is unique because it involves a low-energy impact without prior joint issues, which is an uncommon mechanism for this severe injury. It contributes to the existing literature by demonstrating a favorable early outcome following surgical repair and rehabilitation, though continued monitoring for complications like heterotopic ossification is necessary.

## Case presentation

**Figure 1:**
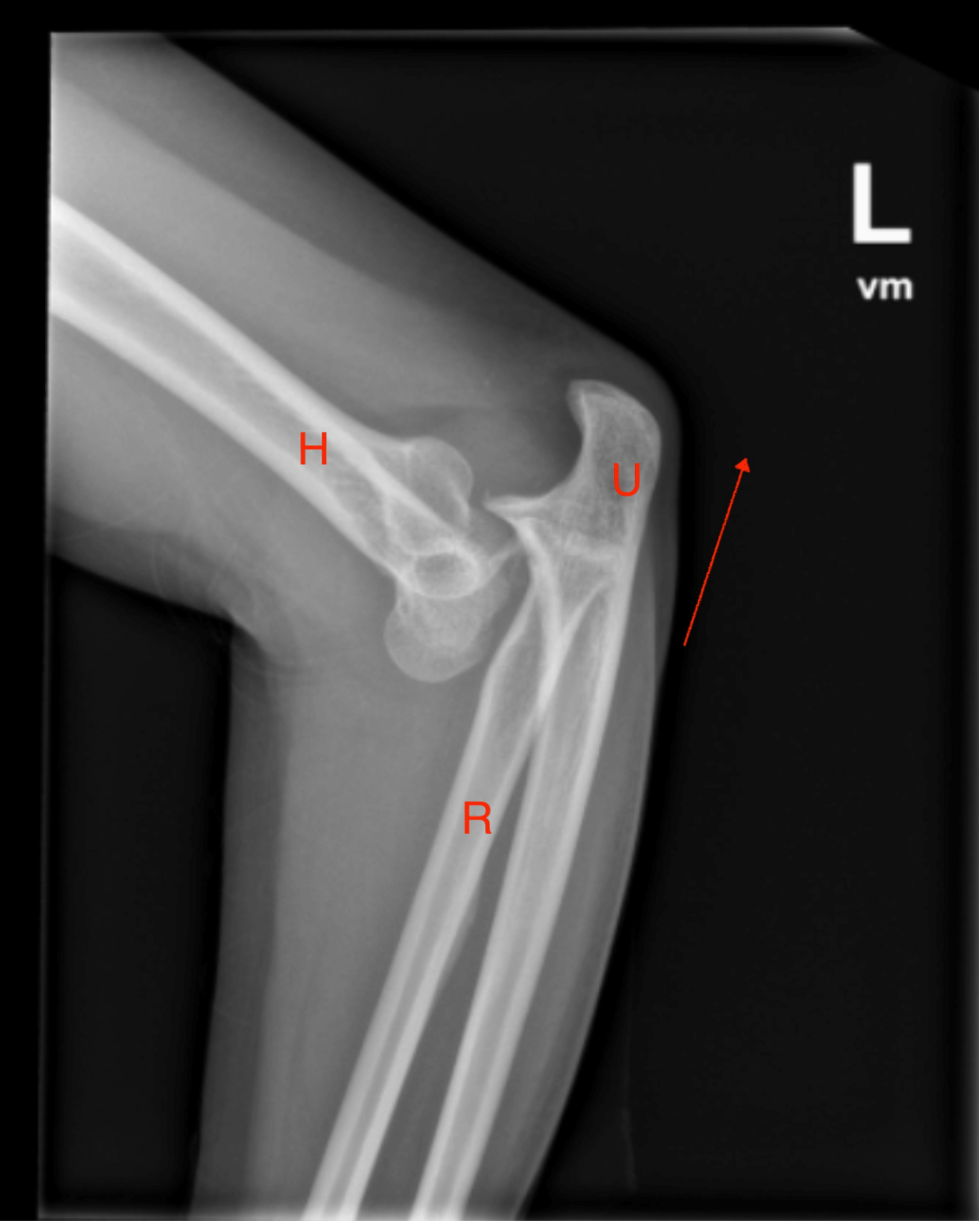
An X-ray of the left elbow before reduction showing posterolateral dislocation in the direction of the arrow. H: humerus; R: radius; U: ulna

A 51-year-old female patient with no significant past medical history and no family history of similar events presented after a fall onto her outstretched arm in her bathroom with resultant left elbow pain, swelling, and disfigurement. She was seen and evaluated in the emergency room shortly after and reduced. Imaging suggested a radial head fracture initially (Figure 1). She has limited nonsteroidal anti-inflammatory use due to a history of allergy to ibuprofen and aspirin. She was prescribed Norco 5 mg for pain management at the time. She then returned for a second opinion due to continued pain. She was evaluated by an orthopedic surgeon, who recommended surgical intervention.

**Figure 2:**
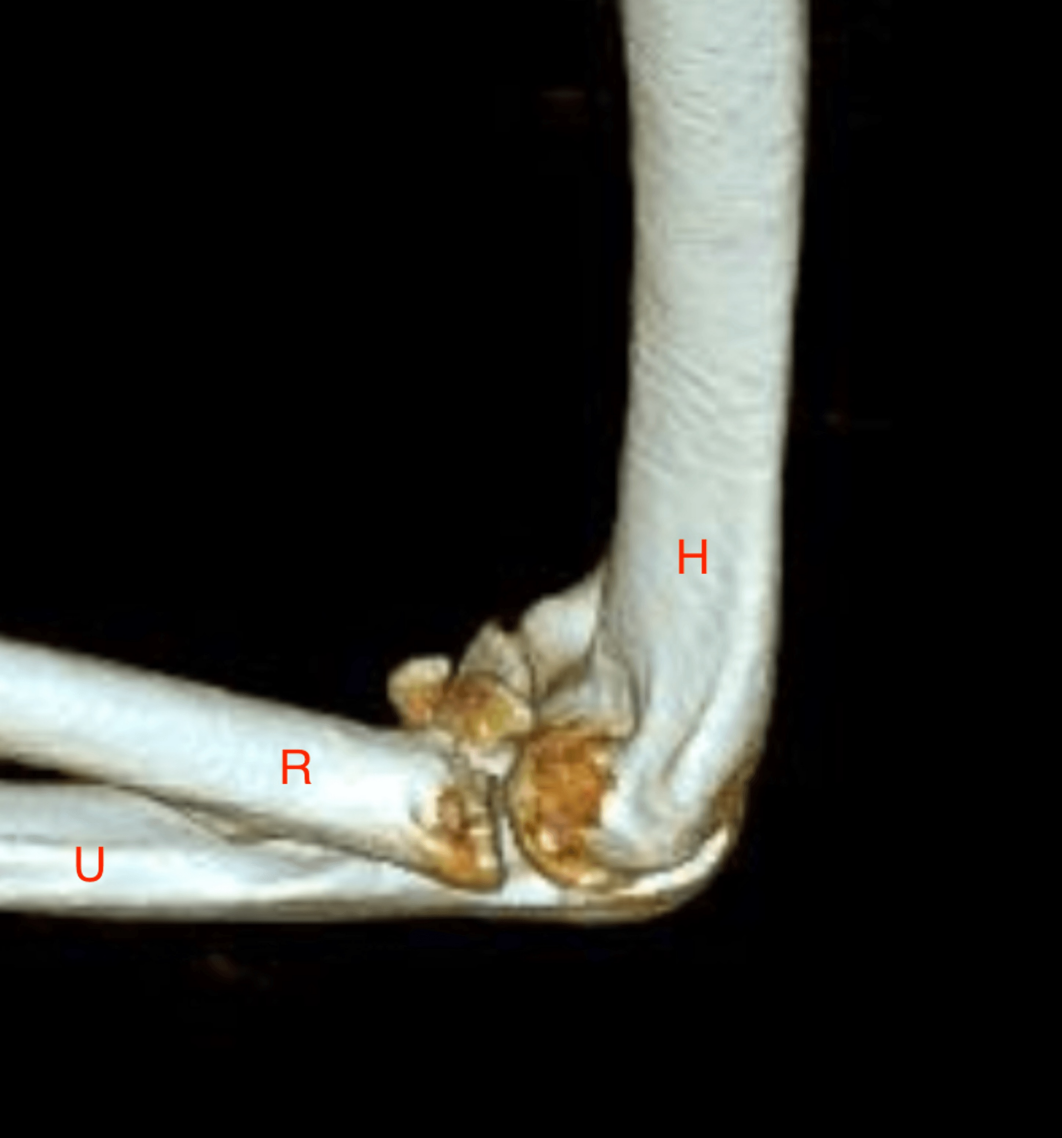
Computed tomography (CT) of the left elbow showing a comminuted fracture (highlighted in yellow). H: humerus; R: radius; U: ulna

On presentation, the patient was noted to be in a long-arm splint with a good digital range of motion (ROM). The CT scan confirmed a severely comminuted radial head fracture and anterior coronoid fracture and findings suggestive of ligamentous injury of both the radial collateral and ulnar collateral ligaments (Figure 2).

The patient underwent open reduction and internal fixation (ORIF) with the following procedures: evacuation of hematoma, irrigation of the joint, radial head replacement, and repair of the LCL and torn extensor tendon. Intraoperatively, a complete rupture of the radial collateral ligament and a severely comminuted radial head fracture were confirmed.

**Figure 3:**
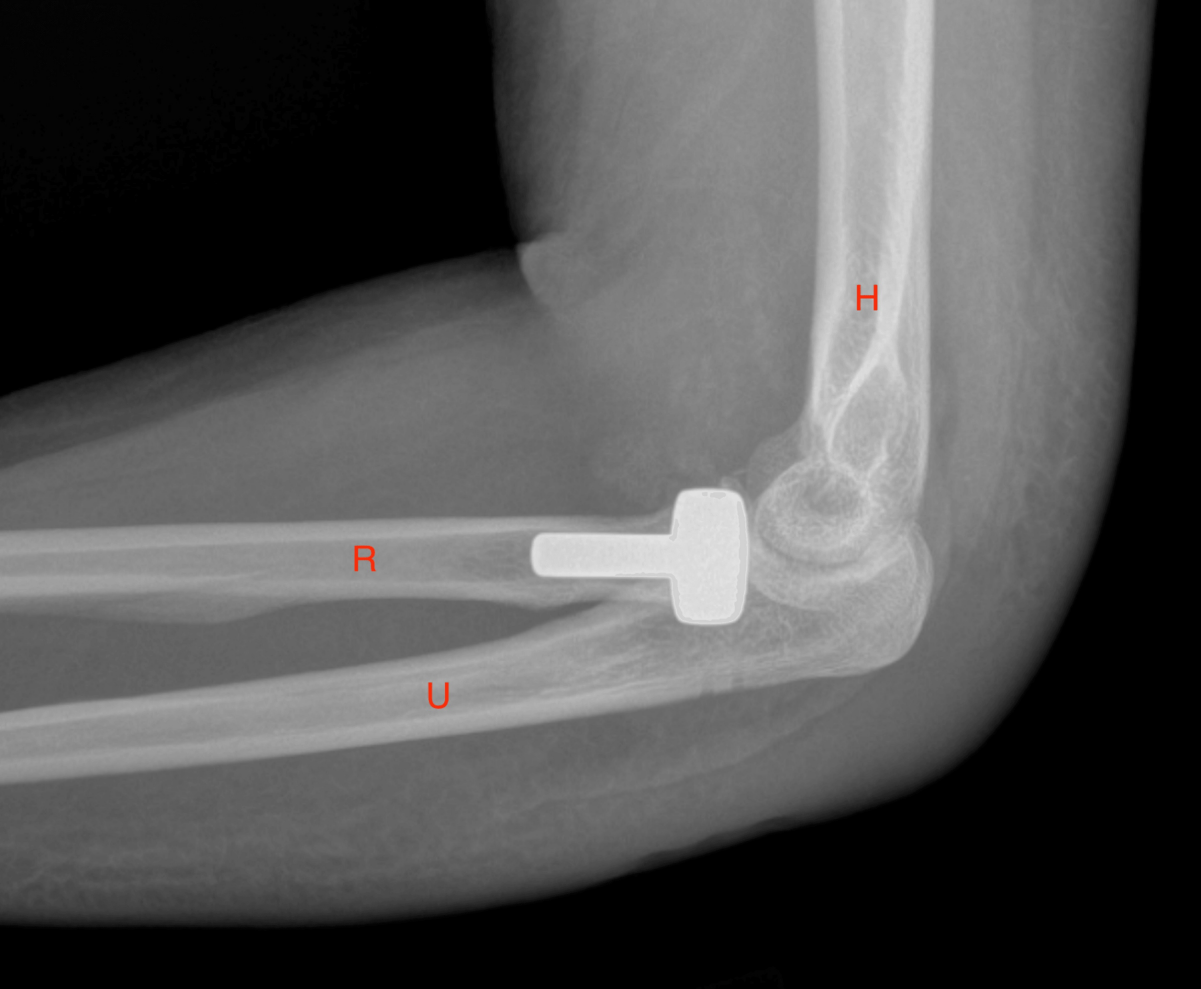
An X-ray of the left elbow after surgery with radial head replacement intact. H: humerus; R: radius; U: ulna

On postoperative day 11, the patient reported muscle aches and back pain. The examination noted significant elbow edema, a clean, dry, and intact incision, and limited ROM with a passive elbow ROM from 45° to 105° and 20° of pronation and supination. Radiographs showed a prosthetic radial head replacement and repair of the coronoid (Figure 3). She was instructed to continue wearing a thermoplastic long-arm splint and to start home exercises targeted at improving her ROM. Naproxen 500 mg twice daily was prescribed for 10 days to manage inflammation. 

**Figure 4:**
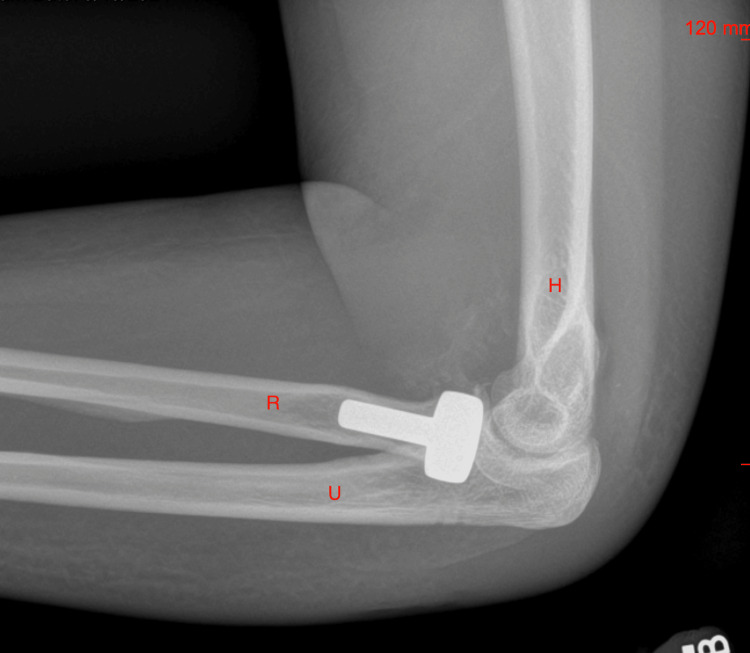
An X-ray of the left elbow on postoperative day 15. H: humerus; R: radius; U: ulna

At her follow-up on postop day 15, her passive ROM had improved to 40° to 115° elbow flexion, 40° pronation, and 50° supination. Radiographs demonstrated a congruent and well-reduced elbow but raised concerns about possible early heterotopic ossification due to opacities in the anterior joint (Figure 4). She was advised to continue naproxen therapy and active assistive ROM exercises at home, with a follow-up in one week.

On postoperative day 21, the patient remained in a splint, performing home exercises, and continued with naproxen therapy. Physical examination showed an unchanged ROM (40° to 115° flexion, 40° pronation, 50° supination). Formal rehabilitation was introduced after this visit, and the patient’s recovery is ongoing, with continued focus on ROM and close monitoring for complications such as heterotopic ossification.

## Discussion

This case adds to the body of literature on terrible triad injuries by showcasing the presentation in a previously healthy individual with no past significant medical history diagnoses. This further demonstrates the importance of timely surgical intervention and early rehabilitation to promote optimal outcomes.

With radial head fractures, there can be associated capitellar chondral defects and loose bodies. Making these findings early can better help to tailor treatment routes in a timely fashion [4].

In complex elbow dislocations, there are 10% to 20% of cases with additional ipsilateral upper extremity injuries. Therefore, a CT is recommended for fracture pattern, displacement, comminution, intra-articular osseous fragments, and surgical planning [4,11].

Nonsurgical care can be utilized if the elbow is minimally or nondisplaced, stable, has a 30- to 45-degree ROM with full extension, and has concentric ulnohumeral and radiocapitellar joints on imaging. Management for suitable patients involves brief immobilization in 90- to 120-degree flexion for the first two weeks but no longer than three weeks. Supervised ROM exercises should be encouraged with limitation of flexion and extension to 30 degrees during weeks one and two with strengthening conditioning only after six weeks and regaining full ROM [4].

With surgery, terrible triad injuries with isolated valgus instability may not require repair of the MCL [4]. Though not seen in this patient, elbow hinges can be placed, which have a contribution to the patient’s recovery prognosis. Comparing external and internal hinged fixators, internal ones have fewer complications and secondary surgeries but are more expensive [7]. Positioning of immobilization of the elbow can also be helpful in various cases. When there is an intact MCL, immobilization in full pronation helps to protect the LCL. Similarly, full supination protects the MCL, and neutral positioning should be used if there is a combined MCL and LCL injury. The postoperative ROM goal is 30 to 130 degrees, with active supervised gentle ROM encouraged starting days two to five to reduce elbow stiffness-similarly to this patient. Early pronation and supination should be limited to protect the collateral ligament repair with good pain control to augment the therapy. Four to eight weeks after surgery, conditioning can be initiated to strengthen the elbow flexors and extensors. Then after six to eight weeks, unsupervised home exercise can be started and continued up to six months as needed. Although the patient regained functional ROM, she continued to report mild discomfort with weight-bearing activities, which is consistent with known post-surgical stiffness in terrible triad injuries. It is important to note that there are currently no recommendations available to help prevent the onset of posttraumatic osteoarthritis in the long term [4]. Stability should be prioritized over motion due to the ability of capsular excision to restore movement, as instability can cause irreversible damage to the articular surface [12].

Should imaging be necessary during this time, overhead gravity-assisted flexion and extension can be utilized, which also protects medial and lateral soft tissues. This aids in identifying residual soft-tissue laxity, or drop sign, on lateral radiographs [4].

Multiple options, including hinged braces and thermoplastic splints, are available for postoperative bracing. Hinged braces can be expensive and difficult to fit. Thermoplastic splints allow immobilization in preferred positioning in flexion and extension with pronation and supination to specific degrees while being flexible to remove for rehab [4]. In this patient, the thermoplastic splint was chosen to encourage early mobilization and physical therapy.

Though not seen in this patient, the terrible triad of the elbow can present in different variations, especially with injury resulting from high-energy mechanisms. Therefore, a thorough physical examination of the entire upper extremity followed by appropriate imaging diagnostic studies is critical [13]. Some variants involve osseous disruption and/or chondral injuries, while also presenting only two of the three classical elements of elbow dislocation, coronoid fracture, and radial head fracture. There could also be capitellum fracture, radial neck fracture, coronoid chondral injury, and medial condyle fracture [13].

Concomitant injuries can also occur, most commonly olecranon fractures, but do not exclude Essex-Lopresti, triceps tendon avulsion, and carpal fracture-dislocation. Therefore, detailed attention to the entire upper extremity is critical, especially in the presence of a large transfer of energy from high-energy impacts [13].

Following terrible triad injuries, complications arise in around 30% of cases, with commonly seen complications including heterotopic ossification (11%), stiffness (1.7%), neurovascular injury-ulnar (2.6%), persistent instability, pain, posttraumatic arthritis, and revision surgery (7.9%) [7]. 

## Conclusions

This case underscores the need for early identification, prompt surgical management, and interprofessional collaboration in treating terrible triad injuries. Continued investigation through case series could help refine rehabilitation protocols and help ensure that even rare elbow injuries can be managed effectively in sports medicine and orthopedic practices.

Recommendations include imaging before and after the reduction of the elbow and CT with 3D reconstruction. If there are more than three fragments, arthroplasty and resection can be considered in addition to the bone quality of the patient. In younger patients, fixation can be a more viable option. Early ROM is important; however, a significant challenge is residual elbow instability. While the patient’s ROM at three weeks (40°-115° flexion) suggests good early recovery, further follow-up is needed to determine if full functional restoration is achieved. Further, mild discomfort with weight-bearing persisted, highlighting the need for ongoing rehabilitation and patient-specific pain management strategies. Future studies could examine whether early rehabilitation strategies can further optimize functional outcomes in low-energy terrible triad injuries.
